# Effects of Extract from Cole Pollen on Lipid Metabolism in Experimental Hyperlipidemic Rats

**DOI:** 10.1155/2014/982498

**Published:** 2014-07-24

**Authors:** Yue Geng, Wen-li Tu, Jing-jing Zhang, Liang Zhang, Jian Zhang

**Affiliations:** Provincial Key Laboratory of Animal Resistance Research, College of Life Science, Shandong Normal University, Jinan 250014, China

## Abstract

In order to evaluate the effects of extract by SCE (supercritical carbon dioxide extraction) from cole pollen on lipid metabolism in hyperlipidemic rats, the experimental hyperlipidemic rats were established by providing with high fat diets, and randomized into six groups. After four weeks of perfusion diets into stomach, the rats were executed, and lipid levels of serum and hepatic tissue were detected. The serum levels of TC and TG were significantly lower in the pollen extract groups and MC group than in HFC group. Hepatic TC levels were decreased in rats fed pollen extract and lovastatin compared with HFC group. A higher concentration of HDL-C and apoAI in hepatic tissue was measured after intake of the pollen extract compared to the HFC group (*P* < 0.05). LCAT activity in serum of pollen extract groups was significantly higher than that in HFC group, and also HMG-CoA reductase showed decreasing tendency in pollen extract groups. The contents of DHA in pollen extract groups were found higher than those in HFC group. Cole pollen extract enriched in alpha-linolenic acid is likely to be a novel source of ALA which is probably responsible for favorable lipid changes through promoting transportation, excretion, and metabolism of cholesterol in hepatic tissue and serum.

## 1. Introduction 

Hyperlipidemia is considered to be a primary risk factor in development of atherosclerosis which is mainly related to dietary habit, so it is important to prevent hyperlipidemia in the event of curing coronary heart disease (CHD). It has long been recognized that n-3 polyunsaturated fatty acids such as EPA (eicosapentaenoic acid) and DHA (docosahexaenoic Acid) exhibit a beneficial effect on reducing CHD and atherosclerosis (AS). As the precursor of n-3 fatty acids, ALA (alpha linolenic acid) is associated with cardioprotective effect [1] and inversely related to the prevalence ratio of CHD [2]. Results from 10 years of follow-up suggest that high intake of ALA reduces the risk of fatal ischemic heart disease [3]. In secondary prevention trial, de Lorgeril reported significant reduction of cardiovascular mortality in the groups assigned to consume Mediterranean ALA-rich diet compared to the usual [4].

It is well known that China is most abundant in pollen source in the world. Our previous studies showed that a wild variety of polyunsaturated acids are found in plant pollen especially in* Brassica campestris* L. and* Zea mays* which contain high level of ALA and reasonable ratio of saturated acid : unsaturated acid [5]. In order to encourage the use of pollen as possible hypolipidemic nature product, we conducted the study to examine whether extract from cole pollen has lipid-lowing effects on rats with supercritical CO_2_ extraction technique (SCE) which have the great benefits of high rate of extract, low energy consumption, and being pollution free.

## 2. Materials and Methods

### 2.1. Preparation of Experimental Extract

Cole pollen (*Brassica campestris* L.) has been processed with SCE technique by Huali pumping company in Hangzhou. The technology for the preparation of the experimental extract has to be optimized at 55°C under 30 MPa pressure for 2 hours, while 45°C and 14 Mpa as well as 40°C and 6 MPa of isolation pots I and II, respectively. Extract from pot I was employed in the experiment.

### 2.2. Animal

Sixty male Wistar mice were purchased in the 140 ± 20 g weight range from the Shandong University of Traditional Chinese Medicine, and then experimental hyperlipidemic rats were set up according to Miao [6]. After 1 week of acclimatization, rats were randomly divided into six groups: including normal control group (NC), high fat control group (HFC), medicinal control group (MC), lo-pollen extraction group (LPE), mid-pollen extraction group (MPE), and hi-pollen extraction group (HPE). NC group was fed a conventional basal diet, while others were supplied with semisynthetic diets; besides MC group that received 10 mg/kg*·*d lovastatin, LFA, MFA, and HFA groups were additionally given pollen extract at the dose of 0.4 mg/kg*·*d, 1.0 mg/kg*·*d, and 2.0 mg/kg*·*d, respectively. The control semisynthetic diets used in these groups contained 2% cholesterol, 10% lard, 0.2% methylthiouracil, and 87.8% basal diet. The rats were perfused with medicine or pollen extract into stomach corresponding to its assignment, weighted once a week, and allowed to drink ad libitum. At 28 days of life after 12-hour fast, the rats were anesthetized by ether. Blood samples were collected from abdominal aorta with EDTA as anticoagulant and livers were harvested and weighted immediately and then stored at −70°C for analysis.

### 2.3. Laboratory Methods

#### 2.3.1. Blood and Live Lipids Measurements

Plasma TG (total triglyceride), TC (total cholesterol), HDL-C (high-density lipoprotein-cholesterol), and LDL-C (low-density lipoprotein-cholesterol) were determined using the enzyme assay kits purchased from ZhongSheng Beikong Bio-Technology and Science Inc. apoAI (apolipoprotein AI) and apoB (apolipoprotein B) were measured using immunoturbidimetric assay kits supported by RongSheng Bio-Technology Inc. The livers were homogenized with a Potter-Elvehjem glass-Teflon homogenizer in aquiferous solution containing 0.9% NaCl. The homogenate was centrifuged at 1000 ×g for 10 min at 4°C and the supernatant was taken for analysis of TC, HDL-C, LDL-C, apoAI, and apoB as for the plasma samples. Free cholesterol of liver was determined using enzyme assay kits purchased from LanYi Bio-Technology Inc.

#### 2.3.2. Determination of Activity of LCAT and HMG-CoA Reductase

Serum LCAT (lecithin cholesterol acyl transferase) was measured by a simple method modified by Jiang et al. [7] and its activity was expressed as micromol of cholesteryl ester generated per hour per liter of serum. HMG-CoA (3-hydroxy-3-methyl-glutaryl-CoA) reductase were isolated from hepatic microsomes by preparative ultracentrifugation and determined as previously reported [8]. Reductase activity was expressed as a micromol of mevalonate produced per mg of protein per minute.

#### 2.3.3. Assay of Fatty Acids Composition

The samples were either directly obtained from cole pollen extract by SCE or extracted from hepatic tissue samples with chloroform/methanol according to Folch et al. [9]. Fatty acid methyl esters were obtained according to the Chinese National Standard “Animal and vegetable fats and oils—preparation of methyl esters of fatty acids” (GB/T 17376-1998). The methyl esters were then analyzed by GC/MS using Rtx-5MS elastic capillary silica column (15 m × 0.25 m) and helium as carrier gas. Procedure of measurement was implemented as previously reported [5]. The components of fatty acids were determined on the basis of searching NIST Library and adopted unitary area to calculate relative concentration.

### 2.4. Statistic Analysis 

The values are expressed as mean ± SD. Data were evaluated with independent *t*-test. All statistical analyses were performed with the statistical program SPSS9.0 for Windows.

## 3. Results 

### 3.1. Fatty Acid Composition of Pollen Extract

From Table 1, results showed the relative content of ALA in extract of pot I had reached 71.25% which was higher than that in perilla oil [10]. Meanwhile there was only 14.04% in pot II. Weight gain and liver index of rats showed no significant differences among experimental groups (data not shown), indicating that little effects were exerted by pollen extract on increasing the body weight of rats.

### 3.2. Changes in Plasma and Liver Lipids Levels

As shown in Table 2, a significant decrease in plasma triacylglycerol after intake of pollen extract was observed as compared with HFC group in our study. TC levels in HDL-C groups rose after intake of lovastatin and pollen extract in comparison with HFC group, but the results were not significant difference except LPE group. The tendency to decrease LDL-C level was observed without any significant changes between MC, LPE, and MPE groups. None of extract pollen groups showed effect on increasing apoAI, whereas there was an upward trend in apoB in pollen extract groups compared with HFC group, especially in HPE group.

**Figure 1:**
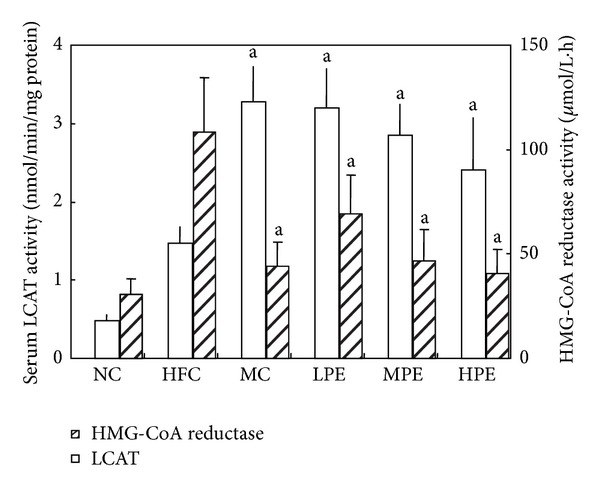
Effect of pollen extraction on LCAT and HMG-CoA reductase of rats (*n* = 10). ^a^ *P* < 0.05 compared to high fat control group.

According to Table 3, MC had lower TC, LDL, and ApoB and higher ApoA1 than the HFC. There were a lot of similarities between MC and pollen extract groups on reducing TC, LDL-C, and apoB levels with exception of little effect on LDL-C with MPE as well as on apoB with HPE group. A significant increase in HDL-C levels occurred within pollen extract groups, indicating that the highest effect was performed by LPE group, followed by MPE and HPE groups. MPE showed higher increasing effect on apoAI than other two pollen extract groups. In addition, LPE and MC groups were observed with a higher reduction of the hepatic free cholesterol levels than MPE and HPE groups compared with HFC, suggesting that the esterifying speed of cholesterol was accelerated by pollen extract in liver. From Figure 1, the general trend in decreasing atherosclerosis index atherosclerosis index [AI = (TC − HDL-C)/HDL-C] with MC and pollen extract groups was notable in serum and liver in comparison with HFC group.

### 3.3. LCAT and HMG-CoA Reductase Activity

In Figure 1, MC and pollen extract groups were likely to increase activity of LCAT which utilizes fatty acyl moiety of phosphatidylcholine to convert cholesterol to cholesteryl ester and accordingly play an important role in maintaining cholesterol balance. Moreover, HMG-CoA reductase activity which is a rate-controlling enzyme of cholesterol biosynthesis significantly declined in MC and pollen extract groups compared with HFC group. These results suggested that pollen extract is good for improving cholesterol transport from plasma to liver, increasing esterification effect and clearance speed when cholesterol was carried from tissue to liver after pollen diet administration. Our results have something in common with early report from Du et al. [10] that ALA affluent diets suppress activity of HMG-CoA reductase that resulted in decreasing biosynthesis of cholesterol in vivo. These studies might closely relate to explaining the low effects of pollen extract on serum cholesterol.

### 3.4. Fatty acid Composition in Liver

The changes of the liver fatty acid composition were demonstrated in Table 4. There were significant differences in oleic levels between NC group and other groups ingesting high fat diet (because high fat diet had 10% lard), also arachidonic acid and DHA were significantly higher in NC group than others. None of the high fat dietary test groups resulted in significant changes in concentration of saturated acids such as palm acid, stearic acid, and linoleic acid as well as SFA/PUFA ratio, while the DHA levels were significantly increased in MC and pollen extract groups compared with HFC group.

## 4. Discussion

This study demonstrates that ALA is the principal component of cole pollen extract by SCE which markedly decreased triglyceride and total cholesterol concentration of plasma and liver. The results suggested that lipid-lowing effects are achieved through changes in lipoprotein and lipid metabolic enzyme like LCAT and HMG-CoA reductase, moreover, alteration fatty acid component of liver.

So far no strong evidences have proved that ALA has direct effects on CHD [11]. It has been clearly investigated that ALA converts to EPA and DHA to a low extent through desaturation and elongation reaction which is limited by Δ6 desaturase [12–14]. However, some previous studies reported ALA that contributed to meet demand of DHA accretion in vivo similar to DHA in the different tissue [15], and effect on hypolipidemia was relatively to increasing levels of EPA and DHA [16–18]. These results are consistent with our observation that the level of DHA has increased in pollen extract groups compared with HFC group. The reason that different ratios of ALA : LA lead to various biosynthesis outcomes might illustrate the fact we studied that there is no difference among three doses of pollen extract groups [11, 15, 19, 20].

These changes in plasma lipid levels are similar to those previously studied by Kim et al. [10, 16, 17, 21], who verified that either ALA-enriched plant oils such as linseed and perilla oils or fish oil have effect on improving hyperlipidemia. It was investigated that ALA suppressed hepatic lipogenic enzymes such as fatty acid synthase and glucose-6-phosphate dehydrogenase [17] and prefer to oxidize rather than esterify as substrate in liver and in turn reduce the TG synthesis and activity of triacylglycerols [22]. All these effects apparently act as a possible mechanism for pollen extract to decline triacylglycerol level.

Although we observed significant decreasing effect on cholesterol in serum and lipid by pollen extract in our study, levels of serum LDL-C were not influenced during the trial, even higher in HPE group in contrast to HFC group. This result is comparable to early researches that ALA-rich perilla was capable of lowing cholesterol concentration [10, 16, 18, 21], while little effect was available to decrease LDL-C [23–25]. As one of the elements of lipoprotein, changes of fatty acid component lead to alteration of physical property and chemical structure of lipoprotein particle so as to impose effects on metabolism of cholesterol and lipoprotein. PUFAs lower the melting temperature of LDL cholesteryl ester [18, 26] and influence protein subpopulation distribution [27]. Moreover Sørensen et al. reported that PUFAs decrease LDL density, increase LDL particle size which reduces the potential oxidization, and in turn decline the risk of AS [23, 24]. Also PUFAs rise LDLR activity and affinity of LDL with LDL receptor, elevate the ability of LDL uptake in the receptor-dependent manner, and accelerate the clearance speed of cholesterol [28]. Meanwhile PUFAs increase levels of cholesteryl ester and lysophosphatidylcholine which induced changes of structure of HDL particle tending towards fluidity and density as favorable carrier during reverse cholesterol transport [29].

As the signal of gene expression regulator, ALA is closely linked to cholesterol metabolism. Either fatty acid alone or synergistically with cholesterol reduces sterol regulatory elements (SRE) expression through alteration mature type of sterol regulatory elements binding proteins (SREBP) in the does-dependent manner, and also more degree of unsaturated fatty acid is more decreasing effect acts [30]. Furthermore PUFAs interact with transcription factor-like hepatic nuclear factor 4*α*, *α*, and *β* liver X receptors, peroxisome proliferators-activated receptors, and NF-*κ*B [31–34], which indicate complex mechanism of gene regulation of key enzyme in lipid metabolism [35]. In conclusion, pollen extract promotes transportation, excretion, and metabolism of cholesterol in vivo as a result of augmentative activity of LCAT and HDL-C levels, decreases biosynthesis of cholesterol through inhibition of HMG-CoA reductase activity, and causes increasing level of DHA in liver.

ALA is dietary essential fatty acid which is considered to be one of the substitutes of fish oil with regard to cardioprotection benefit. Pollen extract contains not only the mainly component of ALA but also other kinds of complex substrates including antioxidant; therefore, the lipid-lowing effects on serum and liver are carried out by ALA interacting with other natural products. The results are in accordance with many researches that consuming ALA from diet show preventive effects against cardiovascular disease. The pollen extract exhibits the preponderance potential in some aspect of improving plasma lipids compared to lovastatin; furthermore, compared to traditional ALA-enriched food such as linseed and perilla oil, pollen is prone to be prevalently accepted for well-known as perfect food especially with the extracting technology of SCE which keeps integrative nutrition of the pollen without pollution. Nevertheless some studies reported that ingesting pure ALA is not associated with beneficial effects on cardiovascular heart diseases perhaps owing to various experimental animals and diets source and diversified technology of processing as well as different trial designs [23, 24, 36]. Further research should be conducted in the interest of steady safety usage when ALA is recommended in nutrition and clinical field.

## Figures and Tables

**Table 1:** Fatty acid composition in pollen's extraction of cole pollen [%].

Fatty acid	Isolation pot I	Isolation pot II
C_7:0_	0.06	
C_8:0_		0.05
C_9:0_		0.06
C_10:0_	0.05	
C_12:0_	0.60	0.54
C_14:0_	3.99	16.80
C_16:3n-3_	10.17	0.30
C_16:0_		3.13
C_18:2n-6_	3.99	1.10
C_18:3n-3_	71.25	14.04
C_18:0_	2.27	1.22
C_20:3n-6_	5.48	
C_20:0_	2.13	0.47
C_20_H_42_		46.70
C_21_H_42_		2.67
C_23_H_48_		4.69
C_26_H_54_		4.15
C_44_H_90_		3.95

**Table 2:** Serum lipid and apolipoprotein levels of the experimental rats [*n* = 10].

Group	TG mmol/L	TC mmol/L	HDL-C mmol/L	apoA I mg/L	LDL-C mmol/L	apoB mg/L
NC	0.36 ± 0.18^ab^	0.62 ± 0.24^ab^	0.23 ± 0.03^ab^	34.16 ± 14.36	0.20 ± 0.02^ab^	69.61 ± 6.52^ab^
HFC	1.26 ± 0.25	2.43 ± 0.27	0.42 ± 0.23	41.07 ± 15.89	1.11 ± 0.14	166.87 ± 40.20
MC	0.93 ± 0.08^a^	1.85 ± 0.46^a^	0.64 ± 0.21	40.97 ± 15.23	0.93 ± 0.15	145.68 ± 16.83
LPE	0.91 ± 0.07^a^	1.80 ± 0.30^a^	0.67 ± 0.24^a^	40.56 ± 3.18	0.80 ± 0.20	180.92 ± 37.51^b^
MPE	0.89 ± 0.07^a^	2.08 ± 0.27^a^	0.48 ± 0.20	42.47 ± 14.38	1.03 ± 0.08	179.41 ± 13.13^b^
HPE	0.94 ± 0.15^a^	2.59 ± 1.53	0.62 ± 0.20	39.65 ± 4.97	1.25 ± 0.70	209.72 ± 12.02^b^

^a^ *P* < 0.05, compared with high fat control group, ^b^ *P* < 0.05, compared with medicinal control group.

**Table 3:** Liver lipid and apolipoprotein levels of the experimental rats [*n* = 10].

Group	TC mmol/g∗	HDL-C mmol/g∗	apoA I mg/g∗	LDL-C mmol/g∗	apoB mg/g∗	hepatic FC mmol/kg
NC	0.57 ± 0.25^a^	0.22 ± 0.09	0.35 ± 0.10	0.32 ± 0.14^a^	0.78 ± 0.32^a^	71.28 ± 35.81^a^
HFC	1.38 ± 0.31	0.16 ± 0.07^b^	0.25 ± 0.03^b^	1.08 ± 0.26	1.97 ± 0.46	114.7 ± 30.92
MC	0.80 ± 0.32^a^	0.26 ± 0.15	0.46 ± 0.23^a^	0.47 ± 0.18^a^	0.90 ± 0.59^a^	83.69 ± 25.43^a^
LPE	0.99 ± 0.20^a^	0.40 ± 0.29^ab^	0.38 ± 0.05^a^	0.51 ± 0.20^a^	1.14 ± 0.92^a^	78.94 ± 28.49^a^
MPE	1.08 ± 0.17^a^	0.32 ± 0.12^ab^	0.52 ± 0.29^a^	0.75 ± 0.21	1.01 ± 0.27^a^	96.53 ± 32.72
HPE	0.99 ± 0.22^a^	0.29 ± 0.05^ab^	0.38 ± 0.08^a^	0.70 ± 0.33^a^	1.55 ± 0.75	91.27 ± 57.13

*wet weight, ^a^ *P* < 0.05, compared with high fat control group, ^b^ *P* < 0.05, compared with medicinal control group.

**Table 4:** Fatty acid composition of liver lipids [*n* = 10].

Fatty acid	Group					
	NC	HFC	MC	LPE	MPE	HPE
14:0	0.3 ± 0.1	0.5 ± 0.2	0.3 ± 0.1	0.5 ± 0.2	0.5 ± 0.2	0.8 ± 0.3
15:0	0.1 ± 0.0	0.1 ± 0.0	0.1 ± 0.0	0.1 ± 0.0	0.1 ± 0.0	0.2 ± 0.1
16:0	32.3 ± 2.8	29.7 ± 6.2	29.1 ± 4.0	31.6 ± 6.1	30.4 ± 2.8	30.9 ± 6.7
16:1	0.8 ± 0.6	1.7 ± 1.0	1.3 ± 0.4	1.5 ± 0.3	2.8 ± 0.8	1.4 ± 0.3
17:0	0.6 ± 0.2	0.2 ± 0.1	0.32 ± 0.1	0.1 ± 0.1	0.3 ± 0.1	0.5 ± 0.1
18:0	19.7 ± 2.8	11.7 ± 2.6	13.9 ± 3.3	12.5 ± 3.5	11.2 ± 2.6	11.9 ± 2.1
18:1	8.8 ± 1.7	26.3 ± 7.2	20.7 ± 6.6	21.7 ± 6.8	25.5 ± 6.8	22.6 ± 6.9
18:2n-6	19.7 ± 2.1	23.1 ± 2.5	25.1 ± 2.5	23.5 ± 2.5	23.7 ± 1.9	25.5 ± 1.3
18:3n:6	N.D.	N.D.	N.D.	N.D.	0.02 ± 0.0	0.2 ± 0.0
20:3n-3	N.D.	0.3 ± 0.0	0.4 ± 0.2	0.4 ± 0.2	N.D.	0.2 ± 0.0
20:4n-6	15.0 ± 2.7	5.2 ± 1.3	7.0 ± 3.1	5.8 ± 1.8	4.5 ± 2.1	5.1 ± 1.0
22:6n-3	2.7 ± 0.5	1.0 ± 0.5	1.8 ± 0.7	1.9 ± 0.6	1.4 ± 0.3	1.5 ± 0.7

SFA/PUFA	1.10	0.73	0.76	0.81	0.74	0.79

N.D., not detected.
